# The Galling Truth: Limited Knowledge of Gall-Associated Volatiles in Multitrophic Interactions

**DOI:** 10.3389/fpls.2018.01139

**Published:** 2018-08-07

**Authors:** Renee M. Borges

**Affiliations:** Centre for Ecological Sciences, Indian Institute of Science, Bengaluru, India

**Keywords:** galler, herbivory, parasitoid, multitrophic interactions, plant–galler interactions, plant–insect interactions, volatile organic compounds, VOCs

## Abstract

Galls are the product of enclosed internal herbivory where the gall maker induces a plant structure within which the herbivores complete their development. For successful sustained herbivory, gall makers must (1) suppress the induction of plant defenses in response to herbivory that is usually mediated through the jasmonic acid pathway and involves volatile organic compound (VOC) production, or (2) have mechanisms to cope with herbivory-induced VOCs, or (3) manipulate production of VOCs to their own advantage. Similarly, plants may have mechanisms (1) to avoid VOC suppression or (2) to attract galler enemies such as parasitoids. While research on VOCs involved in plant–herbivore–parasitoid/predator interactions is extensive, this has largely focussed on the impact of piercing, sucking, and chewing external herbivores or their eggs on VOC emissions. Despite the importance of gallers, owing to their damage to many economically valuable plants, the role of volatiles in gall-associated herbivory has been neglected; exceptions include studies on beneficial gallers and their enemies such as those that occur in brood-site pollination mutualisms. This is possibly the consequence of the difficulties inherent with studying internally occurring herbivory. This review examines the evidence for VOCs in galler attraction to host plants, potential VOC suppression by gallers, increased emission from galls and neighboring tissues, attraction of galler enemies, and the role of galler symbionts in VOC production. It suggests a research focus and ways in which studies on galler-associated VOCs can progress from a philatelic approach involving VOC listing toward a more predictive and evolutionary perspective.

## Introduction

**FIGURE 1:**
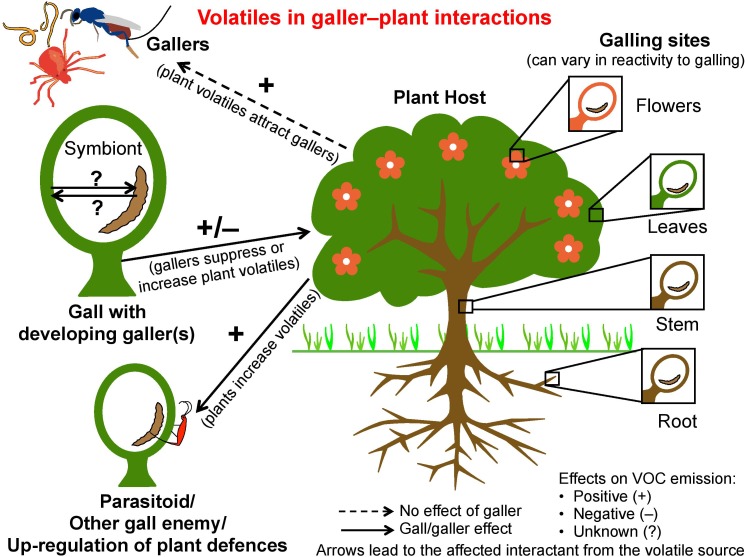
Interactions between gallers and plants by means of volatiles. Arrows point toward the affected interactant from the volatile source. Volatiles may attract gallers to plants; the galler may suppress plant volatile production, or plants may increase volatile production to attract galler enemies or to up-regulate defenses. Symbionts within galls or gallers may affect volatile production.

**FIGURE 2:**
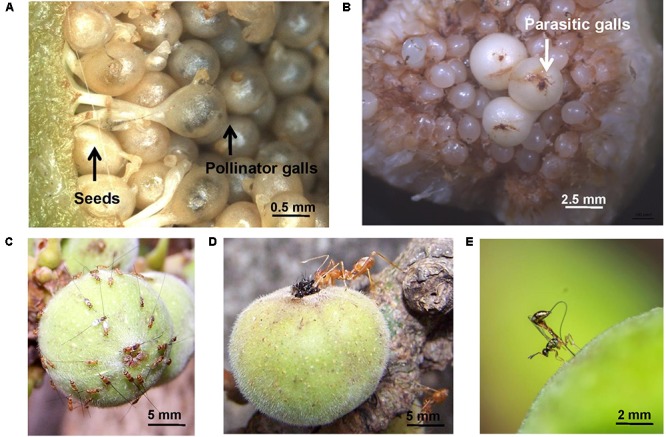
Interactions of plants with beneficial and parasitic gallers. Examples are from the cluster fig *Ficus racemosa* **(A)** seeds and pollinator galls in a syconium. Note remnants of stigma and single developing pollinator in each galled uniovulate flower. **(B)** Large galls of an early-arriving parasitic galler *Sycophaga stratheni* in a syconium; these gallers target tissues of the syconium lumen. **(C)** Aggregations of parasitic gallers *Sycophaga fusca* on a syconium; these gallers are attracted by the syconium volatile blend emitted at pollen-receptive stage. **(D)** The weaver ant *Oecophylla smaragdina* preying upon pollinator gallers *Ceratosolen fusciceps* entering a syconium through the ostiole; ants are attracted by syconial volatiles at pollen-receptive phase. **(E)** Oviposition by parasitoid *Apocrypta* sp. 2 into galls hidden within the syconium; oviposition decision are made using chemosensory features of the ovipositor. Photo credits: **(A,D)** Mahua Ghara and Yuvaraj Ranganathan; **(B**,**C)** Pratibha Yadav; image in **C** is adapted from Yadav et al. (2018) and is reproduced with permission from Springer Nature; **(E)** Nikhil More.

Galls are a classic example of niche construction (Gilbert, 2009) and partly of the extended phenotype of the galling organism (Stone and Cook, 1998). Galls are constructed by gallers in concert with plant tissue that is coerced into gall formation (Favery et al., 2016; Borges, 2017). These hypertrophied tissues provide protection and nutrition for one or more galler generations (Wool and Burstein, 1991). Diverse organisms including viruses, bacteria, fungi, and invertebrates induce galls on plants (Mani, 1964; Raman, 2011; Fernandes and Santos, 2014). Of invertebrates, galling insects are possibly the most diverse and most studied and include gall midges (Diptera: Cecidomyiidae), gall wasps (Hymenoptera: Cynipidae), and aphids (Hemiptera: Aphididae); nematodes and mites are also important. Most galls are an infestation; to be sustained, gallers must suppress or cope with plant defenses such as herbivore-induced plant volatiles (HIPVs) or manipulate them to their own advantage (**Figure 1**). This review is restricted to invertebrate-induced galls, and focuses on the less-examined role of volatiles in galler–plant–galler enemy interactions (**Figure 1**). Beneficial galls occur in some brood-site pollination mutualisms when gallers themselves are pollinators, e.g., fig wasps (Borges, 2016; **Figure 2**). Here the interests of the host-plant and the gallers are aligned, and plants actively signal to their galler pollinators.

## Plant Volatile Organic Compounds (VOCs) That Attract Gallers

Plant tissues rich in meristems are likely most suitable for gall initiation (Carneiro et al., 2017; Silvia and Connor, 2017) and should attract gallers.

### Floral Volatiles

In the fig pollination mutualism, where gallers are pollinators and gall individual flowers at the expense of seeds, a diverse volatile organic compound (VOC) blend attracts agaonid wasp pollinators (Hossaert-McKey et al., 2010; Borges, 2016). These are likely produced by glandular cells in the outer wall of fig syconia (enclosed globular inflorescences) or in bracts surrounding the syconium opening at the pollen-receptive stage (Souza et al., 2015). These blends comprise mostly terpenoids, with some benzenoids and aliphatic compounds (Borges, 2016). In one study, 4-methylanisole was proposed as the major pollinator attractant (Chen et al., 2009). Another study determined that enantiomeric mixtures of some dominant monoterpenes were more attractive to pollinators than others (Chen and Song, 2008). Besides pollinating gallers, most fig syconia also harbor non-pollinating, parasitic galler wasp species (Herre et al., 2008); these arrive for oviposition either very early or much later in the development of the syconium (Segar et al., 2013) attracted by stage-specific VOCs; some species are attracted to the same blends that attract pollinating gallers (Borges et al., 2013; **Figure 2C**), and therefore exploit signals meant for mutualistic gallers.

Sometimes floral VOCs serves as cues for leaf gallers. Floral volatiles in *Salix* are long-distance attractants for leaf-galling sawflies *Pontania proxima* (Kehl et al., 2010). Although the target galling sites are leaves, flowering twigs produce 90 times more VOC quantities than non-flowering twigs suggesting that using floral volatiles as a proxy for leaves may be an efficient host-finding strategy; more flowering than non-flowering plants were galled. In electroantennogram detection (EAD) studies on VOCs from male flowering twigs, compounds absent from vegetative VOC blends, e.g., 1,4-dimethoxybenzene, were strongly detected by sawfly antennae, confirming that such floral compounds may constitute key attractants for leaf gallers. Interestingly, the antennae also responded to green leaf volatiles (GLVs).

### Stem and Leaf Volatiles

Considering the voluminous research on cecidomyiid and cynipid galls, very little work exists on host volatiles as attractants. Volatiles of flowering stems of the herbaceous perennial *Silphium* (Asteraceae) attracted the cynipid gall wasp *Antistrophus rufus* (Tooker et al., 2005). A monoterpene blend consisting of a racemic mixture of α-pinene and β-pinene (+ for both), (+)-limonene, and (-) camphene served as principal attractants for ovipositing females (Tooker et al., 2005). The compound ratios in the blend must be crucial since these monoterpenes are present in sympatric *Silphium* species to which the cynipids are not attracted. Male cynipid wasps use parts of this same blend to locate females within galled stems indicating that host volatiles are employed as mate location cues (Tooker et al., 2002; Tooker and Hanks, 2004) as in several other non-galling phytophagous insects (Xu and Turlings, 2018). The cynipid chestnut gall wasp *Dryocosmus kuriphilus* was attracted to a GLV blend from *Castanea* stems 60–120 min after damage, and failed to be attracted to intact stems (Germinara et al., 2011). All these compounds were detected by wasp antennae. C6 volatiles from young apple leaves were major attractants, eliciting EAD responses in the apple cecidomyiid midge *Dasineura mali* (Anfora et al., 2005). Female orange wheat blossom cecidomyiid midges *Sitodiplosis mosellana* were attracted by key compounds, e.g., (*Z*)-3-hexenyl acetate, acetophenone, and 3-carene, present in minor proportions in the overall volatile profile of wheat panicles (Birkett et al., 2004). The African rice gall cecidomyiid midge *Orseolia oryzivora* preferred volatiles from uninfested plants while those from infested plants served as repellents; the major difference between these volatile profiles was a considerable increase in the HIPV (*E*)-β-caryophyllene (Ogah et al., 2017).

### Root Volatiles

For root-knot nematodes, CO_2_ seems to be the most important attractant released by actively respiring roots (Rasmann et al., 2012); there is scant information on other root volatiles that serve as galler/plant parasitic nematode attractants in the absence of plant damage (Rasmann et al., 2012; Johnson and Rasmann, 2015). Low concentrations of lauric or dodecanoic acid attract root-knot nematodes while this VOC is repellent at high levels (Dong et al., 2014).

## Impact of Gall Makers on Plant Volatiles

### Suppression of Volatile Production

A successful galling strategy may require that gall makers suppress the induction of plant defenses (Kant et al., 2015), since a galler must have prolonged residence within the plant. Since many plant-induced defenses involve activation of the jasmonic acid (JA) pathway, which also often results in volatile release, it is therefore not surprising that VOC production is often suppressed during galling. A meta-analysis of secondary metabolites that are up-regulated on gall induction found that, unlike other metabolites, volatiles were usually unaffected (Hall et al., 2017). For example, goldenrod plants *Solidago altissima* showed no increase in VOC emission after attack by galling flies *Eurosta solidagnis* or galling moths *Gnorimoschema gallaesolidaginis* (Tooker et al., 2008), as also in Japanese elms attacked by galling aphids (Takei et al., 2015). Furthermore, infestation by *E. solidagnis* suppressed HIPVs in response to subsequent herbivory by generalist caterpillars (Tooker et al., 2008). Consistent with JA suppression, galls accumulated salicylic acid (SA) instead (Tooker et al., 2008). Tooker and De Moraes (2008) speculate that gallers are adapted to suppress JA, since JA inhibits plant growth hormones such as auxin and also cytokinins, both of which must be locally up-regulated in gall formation (Tooker and Helms, 2014). Whether gallers can suppress ethylene production which could impact VOC production (Broekgaarden et al., 2015) is unknown. Some insect gallers may synthesize phytohormones, e.g., auxin (Bailey et al., 2015); this may impact JA synthesis as suggested by cross-talk between auxin and JA observed in many plant-associated bacteria and fungi (Berens et al., 2017). Gallers may succeed in suppressing plant defenses by deploying effector molecules (Zhao et al., 2015) among which are ATP-hydrolysing enzymes, calcium-binding proteins, and ubiquitin ligases (Giron et al., 2016; Guiguet et al., 2016; Nabity, 2016).

Five non-mutually exclusive mechanisms have been suggested for the absence of increased VOC emission after galling (Tooker et al., 2008). Besides the SA up-regulation mentioned above, they include (a) avoidance of galler detection, (b) targetting relatively non-reactive tissues, e.g., stems, (c) depletion by galler larvae of plant resources needed for VOC production, and (d) active suppression of host-plant defense (e.g., Nyman and Julkunen-Tiitto, 2000).

### Increase in Volatiles After Galling

While gallers often suppress VOCs, increased VOC production in and around galled tissues may occur. In flower galls produced by the dipteran *Myopites stylatus* on the woody fleabane *Dittrichia viscosa* (Asteraceae), emission of the phenylpropene compound estragole, an isomer of anethole, increased six times compared to ungalled flowers (Santos et al., 2016). A moderate increase in anethole was also evident. The terpene eucalyptol (1,8-cineole) was emitted in large quantities only from galls, and was absent from floral scents. However, the gall emission of compounds such as α-pinene, β-pinene, limonene, and linalool was significantly lowered. Terpenes such as α-pinene, limonene, and linalool may have a toxic but hormetic effect on dipterans (Papanastasiou et al., 2017), while estragole and anethole are generally toxic to dipterans (Chang et al., 2009). Eucalyptol emitted by another Asteraceae plant was a repellent and oviposition-deterrent to mosquitoes (Klocke et al., 1987). Many interesting questions arise from these observations on the fleabane–fly interaction. First, the concentrations of the VOCs within the gall are unknown; therefore, whether high concentrations of estragole and anethole are also present within gall tissues is not known. If the gall also contains high amounts of these phenylpropanoids, that are usually toxic to many insects, one may speculate that the galler larvae/pupae are resistant to these toxins. If so, are they being produced by the galler by hijacking plant biochemical machinery to their advantage, so that non-resistant galler enemies such as parasitoids are also deterred? Additionally, is it possible that concentrations of α-pinene, limonene, and linalool are lowered within the gall to non-toxic but hormetic levels under the action of the galler? Is eucalyptol being produced in very high quantities to deter galler enemies such as parasitoids or for its antifungal/antibacterial activities? The parasitoids of this tephritid fly species include eurytomid, eupelmid, pteromalid and torymid wasps, and appear to be attracted by gall and/or host cues (Santos et al., 2016). It is also possible that gallers are unable to manipulate VOC release and that VOC emission is under multifactorial control resulting in unexpected VOC emission patterns.

In another example, the aphid *Baizongia pistaciae* induces galls on the terminal buds of the pistachio *Pistacia palaestina* (Anacardiaceae). Gall tissue extracts contained much higher levels of terpenes, especially α-pinene and limonene, than surrounding ungalled leaves, while leaves accumulated more sesquiterpenes (Rand et al., 2014). The high terpene levels resulted from increased biosynthetic activity within the galls rather than accumulation from surrounding tissue (Rand et al., 2017). High terpene levels within the gall could result from a need for antibacterial/antifungal activity, or to deter parasitoids. Stored and emitted terpenes were also in higher concentrations in galls formed by the aphid *Slavum wertheimae* on the lateral buds of *Pistacia atlantica* (Rostás et al., 2013). Concentrations of three terpenes, i.e., α-pinene, limonene, and sabinene, were much higher compared to others. The principal galler enemies in this study were mammalian herbivores, i.e., goats, that were reluctant to consume galls or food pellets to which these three terpenes were added in biologically relevant concentrations. While the authors speculate that terpenes were largely responsible for the feeding deterrence, they admit that the high gall tannin concentrations may also have deterrent effects.

In the oak *Quercus robur*, cynipid galls do not have altered VOC emissions but affect emissions of neighboring portions of gall-bearing leaves (Jiang et al., 2018). The change in leaf emission depends on whether the galler attacks major veins or intercostal areas. Major vein galls resulted in more GLV production and less terpenes from neighboring tissues, while galls in undifferentiated parenchyma of intercostal areas resulted in much more terpene and benzenoid production. Notably both types of galls caused increased α-pinene and limonene emission, while the intercostal tissue galls also induced the emission of other monoterpenes such as linalool, camphene, β-myrcene, and the sesquiterpene β-bergamotene that were not produced by ungalled leaves. Galls also produced far less isoprene than ungalled leaves. Therefore, in this example also, monoterpene production was most affected. Similarly, an increase in α-pinene and limonene emission occurred in galls induced by psyllids on *Schinus polygamus* (Anacardiaceae) (Damasceno et al., 2010); C6 volatiles increased in neighboring leaf portions bearing the galls.

In *S. altissima* attacked by the rosette gall-midge *Rhopalomyia solidaginis*, emissions of terpenes such as copaene and β-pinene increased post-galling (Uesugi et al., 2016); these attracted herbivorous beetles whose presence facilitated galler performance, suggesting that VOC emission patterns must be viewed in an integrated manner.

## Plant or Gall Volatiles That Attract Galler Enemies

Herbivore-induced plant volatiles used by parasitoids in host location have been largely investigated for externally feeding chewing, piercing, and sucking herbivores (Aartsma et al., 2017), where herbivore feeding mode appears an accurate predictor of the HIPV blend (Danner et al., 2017). Surprisingly, there is almost no work on VOCs attracting parasitoids to galls. While Borges et al. (2013) examined changes in the volatilome during fig syconial development, including stages when a multiplicity of gallers and parasitoids of these gallers are also attracted, here too, the study did not specifically test a set of volatiles on parasitoids. Using an adaptation of weighted gene coexpression network analysis (WGCNA), co-emitted modules of VOCs were detected. Early-arriving gallers triggered the release of GLVs such as (*Z*)-3-hexenyl acetate and (*Z*)-3-hexenol. Later-arriving gallers triggered the release of compounds such as (*E*)-β-ocimene, (*Z*)-β-ocimene, and methyl salicylate in response to galler feeding; these are well known HIPVs and parasitoid attractants in other plant–herbivore systems (Turlings and Erb, 2018). In Y-tube olfactometer experiments, parasitoid wasps were attracted by VOCs of fig syconia containing their galler hosts (Proffit et al., 2007). Such attracted parasitoids make decisions about which galls to parasitise by sampling syconial odors with their probing ovipositor. For the first time, Yadav and Borges (2017) showed that the ovipositor of fig wasp gallers and parasitoids is a volatile sensor, and that it responds both behaviourally as well as with neuronal firing to cues such as CO_2_ and syconial stage-specific volatiles. For parasitoids that need to seek out hidden hosts within galls (**Figure 2E**), the use of cues such as CO_2_ that signal locations of actively respiring galler larvae/pupae are potentially extremely important in successful parasitism. Parasitoids in the fig system may be considered apparent mutualists if they target non-beneficial gallers or control the population of pollinating gallers within fig syconia (Krishnan et al., 2015); therefore, the role of VOCs in maintaining such tritrophic interactions in order to stabilize the core mutualism is likely profound.

Volatiles emitted by fig syconia in pollen-receptive phase to attract galling pollinators are also attractive to predatory ants that eavesdrop on this pollinator signal (Ranganathan and Borges, 2009; **Figure 2D**). These ants are also attracted to syconial volatiles at a later stage when F1 galler wasps and parasitoids exit the syconia; ant attraction toward stage-specific volatiles in Y-tube olfactometer tests is a learnt association based on prior exposure to syconial volatiles (Ranganathan and Borges, 2009). Since ants are important predators of galler and parasitoid fig wasps (Ranganathan et al., 2010; Bain et al., 2014), and are also important in other galling systems (Fernandes et al., 1999), their response to gall-associated volatiles deserves more attention.

Inquilines of gallers are also attracted by volatiles. Goldenrod *Solidago* stems are infested by galls induced by *E. solidaginis* tephritid flies. These flies can infest *S. altissima* and *Solidago gigantea* plants, and in turn their galls are attacked by gall-boring inquiline beetles *Mordellistena convicta*. These beetles were attracted to volatiles associated with galls of their natal host plants, and avoided those of alternate host plants occupied by their galler hosts suggesting that inquiline speciation and subsequent radiation is driven by olfaction (Rhodes et al., 2012). The inquiline wasps *Diaziella yangi* and *Lipothymus* sp. that parasitise the pollinating galler *Eupristina* sp. of *Ficus curtipes* are attracted by 6-methyl-5-hepten-2-one (Gu and Yang, 2013) which is an important volatile in the pollen-receptive scent of the fig syconium (Gu et al., 2012); they showed no attraction to 6-methyl-5-hepten-2-ol which is another important scent constituent.

## Role of Symbionts in Galler–Plant Interactions

Plant scents may be influenced by symbionts. For example, bacteria have been recently implicated in VOC production in floral tissues (Helletsgruber et al., 2017). Bacteria in oral secretions of caterpillars can also suppress JA responses (Wang et al., 2016). Fungal root symbionts can influence above-ground production of HIPVs that attract parasitoids (Rasmann et al., 2017; Simon et al., 2017). Whether microbes are involved in VOC production that is beneficial to the galler or to the plant is as yet unknown (**Figure 1**). However, symbiosis has benefitted many gall-inducing insect lineages such as ambrosia gall midges whose diversification has been aided by fungal symbionts (Joy, 2013). The role of endophytic fungi present in many insect-induced galls is also unknown (Lawson et al., 2014).

## Conclusion

### Moving From Philately to Understanding the Volatilome

While information is still scarce, gall induction may affect the volatilome of plants. Making sense of this effect may be tackled at several levels. (1) Classes of VOCs may have particular relevance at different stages of galler–plant interactions. For example, that many gallers are attracted to non-specific GLVs suggests that GLVs are used as habitat cues rather than host-specific cues (Webster and Cardé, 2017) and enable navigation toward suitable vegetation patches from long distances. (2) Mirroring research on externally feeding herbivores, where HIPVs have been investigated from the egg-laying stage (Hilker and Fatouros, 2015), comparable information is needed in galler–plant–parasitoid/inquiline interactions including stages such as tissue damage by ovipositor probing, egg laying, damage by larval mandibles, and finally gall induction. (3) Metabolic plasticity can occur in host-plant–galler interactions vis-a-vis different gallers and plant genotypes (Uesugi et al., 2013, 2016), resulting in varied responses that must, therefore, be interpreted within the context of the specific interactants. (4) We need to understand correlation networks of VOCs produced by plants under a variety of circumstances. Junker et al. (2017) and Junker (2017) have examined enzymatic and volatile hubs; such efforts may lead to predictions and an understanding of why certain VOCs tend to co-occur, e.g., α-pinene, limonene, and linalool in many of the above-cited examples. (5) Chemists and behavioral ecologists must appreciate the importance of VOCs produced in small quantities; small peaks are often ignored at analytical and testing stages, but these may often contain the real signaling messages (Clavijo McCormick et al., 2014). (6) Since VOC emission is likely an active process controlled by transporters across plant cell membranes (Adebesin et al., 2017), and is not merely controlled by physical processes such as volatility (Borges et al., 2013), the field of plant signaling using volatiles must move toward making predictions about the costs and benefits of VOC production, types of VOCs expected, and their consequences. Only then will the study of volatiles progress from philately to viewing gall-associated volatiles within an ecological and evolutionary framework.

## Author Contributions

The author confirms being the sole contributor of this work and approved it for publication.

## Conflict of Interest Statement

The author declares that the research was conducted in the absence of any commercial or financial relationships that could be construed as a potential conflict of interest.
